# Circulating tumor cells in HER2-positive metastatic breast cancer patients: a valuable prognostic and predictive biomarker

**DOI:** 10.1186/1471-2407-13-202

**Published:** 2013-04-23

**Authors:** Yi Liu, Qian Liu, Tao Wang, Li Bian, Shaohua Zhang, Haixu Hu, Sha Li, Zhiyuan Hu, Shikai Wu, Bing Liu, Zefei Jiang

**Affiliations:** 1 Department of Breast Cancer, Affiliated Hospital of Academy of Military Medical Sciences, No.8 Dongdajie, Beijing, 100071, China; 2 Translational Medicine Center, Laboratory of Oncology, Affiliated Hospital of Academy of Military Medical Sciences, No.8 Dongdajie, Beijing, 100071, China; 3 National Center for Nanoscience and Technology, No.11 ZhongGuanCun BeiYiTiao, Beijing, 100190, China

## Abstract

**Background:**

This study was initiated to investigate the prognostic significance of circulating tumor cell (CTC) enumeration and the predictive value of CTC HER2 expression for efficient anti-HER2 therapy in HER2-positive metastatic breast cancer (MBC) patients.

**Methods:**

Sixty HER2-positive MBC patients were enrolled in the present study. Before the initiation of systemic treatment, CTCs from 7.5 ml of blood were analyzed using the CellSearch system. The progression-free survival (PFS) of the patients was estimated using Kaplan-Meier survival curves.

**Results:**

CTCs were detected in 45% (27/60) of the patients, who had shorter median PFS than those without CTCs (2.5 vs. 7.5 months, *P* = 0.0125). Furthermore, referring to the standard HER2 testing that uses immunohistochemistry (IHC), we proposed a CTC HER2-positive criterion, defined as >30% of CTCs over-expressing HER2. Among patients undergoing anti-HER2 therapy, those with HER2-positive CTCs had longer PFS (8.8 vs. 2.5 months, *P* = 0.002). Among patients with HER2-positive CTCs, the median PFS for those receiving anti-HER2 therapy was significantly longer than those who were not (8.8 vs. 1.5 months, *P* = 0.001). Notably, up to 52% (14/27) of the HER2-positive patients were CTC HER2-negative, and anti-HER2 therapy did not significantly improve the median PFS in these patients (2.5 vs. 0.9 months, *P* = 0.499).

**Conclusions:**

Our findings underscore the necessity of a comprehensive CTC analysis, which may provide valuable prognostic and predictive information for optimizing individually tailored therapies in HER2-positive MBC patients. To test this idea, additional large cohort, multi-center and prospective clinical trials are needed.

## Background

Human epidermal growth factor receptor 2 (HER2) is a 185 kDa transmembrane tyrosine kinase receptor encoded by the HER2 gene on chromosome 17q21. HER2 over-expression or amplification occurs in approximately 20% of all breast cancer patients and is associated with aggressive growth, short survival and poor prognosis [1-3]. HER2 positivity correlates with the clinical outcome of treatment with anti-HER2 agents such as trastuzumab (Herceptin, Genentech, South San Francisco, CA, USA) and lapatinib (Tykerb, GSK, Philadelphia, PA, USA) [4-7]. Therefore, HER2 is considered to be a vital prognostic and predictive factor, and treatment of HER2-positive patients remains one of the great therapeutic challenges in metastatic breast cancer (MBC).

Despite therapeutic advances over the past decades, individually tailored therapeutic regimens for HER2-positve patients remain far from satisfactory. For example, the benefit of single-agent anti-HER2 therapy, in the form of either trastuzumab or lapatinib, is only in the range of ~25% [8]. There are three possible explanations for this phenomenon. First, HER2-positive MBC patients may be divided into subgroups with different prognoses. Second, the initial assessment of HER2-positivity may be inaccurate due to the inherent limitations of traditional methods, including tumor heterogeneity, subjectivity in the interpretation of results or technical limitations such as variability in tissue processing and reagents [9]. Third, previous studies have demonstrated that there are inconsistencies in HER2 expression between primary tumors and their metastases [10,11]. Such inconsistencies indicate that tumor cells are under constant evolvement or clonal selection and that the detected HER2 status may not necessarily reflect the patients’ real-time phenotypes. Therefore, it is critical to discover more precise prognostic marker and real-time methods for HER2 testing to optimize individualized therapeutic regimens for HER2-positive MBC patients.

Circulating tumor cells (CTCs) are cells that shed from the tumor and enter the circulation, a process that is required for cancer metastasis. Considerable efforts have been made to develop technologies for CTC detection and characterization; among these technologies, the CellSearch system (Veridex LLC, Raritan, NJ, USA) is the only one approved by US Food and Drug Administration (FDA) for clinical use in treating MBC [12,13]. CellSearch has a CTC detection cutoff of ≥5 cells/7.5 ml blood, and it has been demonstrated to be an independent prognostic factor for the prediction of progression-free survival (PFS) and the overall survival (OS) of MBC patients [14-17]. In addition to detection, the molecular characterization of CTCs is now recognized as a valuable tool that can provide real-time information to distinguish subgroups of patients who can benefit from certain types of therapy [18,19]. Unfortunately, previous studies failed to illustrate the prognostic value of CTCs in HER2-positive MBC patients using CellSearch [20]. Although recent studies have made great efforts to compare HER2 statuses between tumor tissue and CTCs [18,19,21-29], a satisfactory CTC HER2-positive criterion has not yet been established.

In the present study, we demonstrated that CTC enumeration using a modified cutoff has superior prognostic value for HER2-positive MBC. Furthermore, we proposed that HER2 positivity should be determined by both the HER2 intensity of individual CTCs and the percentage of CTCs with that intensity. Using the criterion defined as >30% of CTCs over-expressing HER2, we found that HER2 expression in CTCs was different from that in tumor tissues, and this expression could significantly improve the response prediction for anti-HER2 therapy.

## Methods

### Study design

Patients who showed clinical and radiological evidence of metastatic breast cancer were randomly enrolled in the present study. Eligible patients were required to have measurable or evaluable disease, with an Eastern Cooperative Oncology Group (ECOG) performance status score of 0 to 3 and a pathology report describing their histological type and nodal status, as well as their estrogen receptor (ER), progesterone receptor (PgR), and HER2 statuses. A patient was considered HER2-positive with an immunohistochemistry (IHC) score of 3+ or a fluorescent in situ hybridization (FISH) ratio of more than 2.2. IHC scores of 0 and 1+ or a FISH ratio of less than 1.8 were considered to be HER2-negative [30].

Before the start of a new line or a new therapy cycle, 10 ml of blood was drawn. An interval of <7 days between the day of blood sampling and the initiation of systemic treatment was required. All treatment decisions for the patients were made according to the National Comprehensive Cancer Network (NCCN) clinical practice guidelines (Breast Cancer V.2.2010) without knowing the patients’ CTC results. Disease status was assessed and categorized according to Response Evaluation Criteria in Solid Tumors (RECIST). After several months of follow-up, the relationship between the quantity and characteristics of CTCs and clinical outcome was analyzed retrospectively.

All patients signed an informed consent to participate in the study, which was approved by the ethics and scientific committees of the Affiliated Hospital of the Academy of Military Medical Sciences.

### Isolation, enumeration and characterization of CTCs

CTC isolation, enumeration and characterization were performed using the CellSearch system, according to the manufacturer’s instructions as described elsewhere [14-17]. Briefly, cells that expressed the epithelial cell adhesion molecule (EpCAM) were immunomagnetically enriched by the semiautomated sample preparation system that was provided with the CellSearch epithelial cell kit. The isolated cells were then automatically labeled with fluorescently tagged monoclonal antibodies specific for leukocytes (CD45-allophycocyanin) and epithelial cells (cytokeratins (CK) 8-, 18- and 19-phycoerythrin) and were stained with the nucleic acid dye 4′,6-diamidino-2-phenylindole (DAPI). HER2 expression in CTCs was assessed by staining the cells with a fluorescein isothiocyanate (FITC)-labeled anti-HER2 antibody (Veridex LLC, Raritan, NJ, USA). The intensity of HER2 expression on CTCs was given a score of 0 (negative), 1+ (weak), 2+ (moderate or questionable), or 3+ (strong) according to criteria described elsewhere [28,29].

According to tissue HER2 positive criterion using immunohistochemistry (IHC) [30], which detects HER2 protein expression with a similar technological principle as immunofluorescent (IF), we set two criteria for HER2 positivity in CTCs: either >30% or >10% of CTCs over-expressing HER2. We then analyzed the clinical outcome of the patients based on these two criteria.

### Statistical analysis

Fisher’s exact test was used to test whether there was a statistically significant difference between the number of patients with a cut-off of 5 CTCs and those with 1 CTC as baseline. PFS was defined as the time elapsed from the initial blood sampling to the documentation of disease progression (according to RECIST) or, if no progression was observed during the follow-up, to the last follow-up visit. Kaplan-Meier survival curves were generated based on the CTC levels at baseline and the HER2 status of CTCs, and the curves were compared using the log-rank test. McNemar’s test was used to determine whether a statistically significant difference existed regarding variations in HER2 status between CTCs and histological results. *P* values <0.05 were considered statistically significant. Analyses were carried out using SAS software version 9.1.3 (SAS Institute Inc., Cary, NC, USA).

## Results

### Patient characteristics and CTC enumeration

From September 2010 to August 2011, 60 HER2-positive MBC patients with a mean age of 49 years (range: 25 to 75 years) were enrolled in the present study. In addition, 11 HER2-negative MBC patients (10 of whom were ER-positive) were enrolled as a control group. The pathological and clinical characteristics of the patients are listed in Table 1 and Additional file 1: Table S1, respectively. As shown in Additional file 2: Table S2, CTCs were detected in 45% (27/60) of the HER2-positive patients, and the CTC count ranged from 1 to 1140 with a mean value of 68. Of the HER2-positive patients with detectable CTCs, 56% (15/27) had a CTC count that ranged from 1 to 4. In contrast, CTCs were detected in 80.0% (8/10) of the ER-positive/HER2-negative patients, and only 25.0% (2/8) of those patients had a CTC count that ranged from 1 to 4. Two different cutoffs were used to divide patients into two groups based on the CTC count at the initial blood draw: the first cutoff was ≥1 CTC, and the other was ≥5 CTCs. There were no statistically significant differences between the two groups in terms of age, histology, status of hormone receptors (HRs) such as ER and PR, metastatic sites and numbers, disease-free survival (DFS), and therapy line.

**Table 1:** Pathological and clinical characteristics of HER2-positive patients at baseline

**Characteristics**	**Total**	**No. patients (%)**		***P***	**No. patients (%)**		***P***
		**CTC Count at baseline**			**CTC Count at baseline**		
		**≥1**	**<1**		**≥5**	**<5**	
**Overall**	60	27 (45.0)	33 (55.0)		12 (20.0)	48 (80.0)	
**Age (years)**							
Mean	48.8	46.6	50.6	0.155	46.6	49.4	0.420
Range	25-75	25-68	33-75		25-58	32-75	
**Histology**							
Ductal	49	24 (49.0)	25 (51.0)	0.130	10 (20.4)	39 (79.6)	0.481
Lobular	3	0 (0.0)	3 (100.0)		0 (0.0)	3 (100.0)	
Others	8	3 (37.5)	5 (62.5)		2 (25.0)	6 (75.0)	
**ER**							
Positive	24	9 (37.5)	15 (62.5)	0.430	4 (16.7)	20 (83.3)	0.746
Negative	36	18 (50.0)	18 (50.0)		8 (22.2)	28 (77.8)	
**PR**							
Positive	22	9 (40.9)	13 (59.1)	0.789	4 (18.2)	18 (81.8)	1.000
Negative	38	18 (47.4)	20 (52.6)		8 (21.1)	30 (78.9)	
**No. of Metastasis**							
1	19	5 (26.3)	14 (73.7)	0.057	1 (5.3)	18 (94.7)	0.082
≥2	41	22 (53.7)	19 (46.3)		11 (26.8)	30 (73.2)	
**Metastatic sites**							
Bone only	4	2 (50.0)	2 (50.0)	0.314	0 (0.0)	4 (100.0)	0.270
Visceral only	7	2 (28.6)	5 (71.4)		1 (14.3)	6 (85.7)	
Bone and visceral	30	18 (60.0)	12 (40.0)		8 (26.7)	22 (73.3)	
**DFS**							
≤12 months	11	3 (27.3)	8 (72.7)	0.306	3 (27.3)	8 (72.7)	0.671
>12 months	39	19 (48.7)	20 (51.3)		7 (17.9)	32 (82.1)	
**Therapy line**							
1	15	7 (46.7)	8 (53.3)	0.470	3 (20.0)	12 (80.0)	0.329
2	9	2 (22.2)	7 (77.8)		1 (11.1)	8 (88.9)	
3	13	7 (53.8)	6 (46.2)		1 (7.7)	12 (92.3)	
≥ 4	23	11 (47.8)	12 (52.2)		7 (30.4)	16 (69.6)	

### Prognostic significance of CTC enumeration

**Figure 1:**
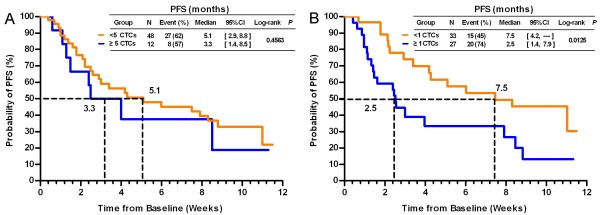
**Kaplan-Meier PFS plots of HER2-positive MBC patients with a cut-off of ≥ 5 (A) and ≥1 (B) CTCs.** PFS was calculated from the time of the baseline blood draw. Coordinates of the dashed lines indicate median survival time.

At the 10-month follow-up visit, 57% (34/60) of the patients exhibited disease progression. Using a cut-off of ≥5 CTCs, no significant difference was found in the median PFS between the two groups (3.3 vs. 5.1 months, *P* = 0.4563, Figure 1A), consistent with a previous report [20]. Considering the lower detection rate of CTCs in HER2-positive patients described above and previously [19,20], we used a lower cut-off and found that patients with ≥1 CTC had a significantly shorter median PFS than those with <1 CTC (2.5 vs. 7.5 months, *P* = 0.0125, Figure 1B). We also analyzed the median PFS for groups divided based on cut-offs of ≥2, ≥3 and ≥4 CTCs, but we found no significant differences (Additional file 3: Figure S1).

### HER2 expression on CTCs

**Figure 2:**
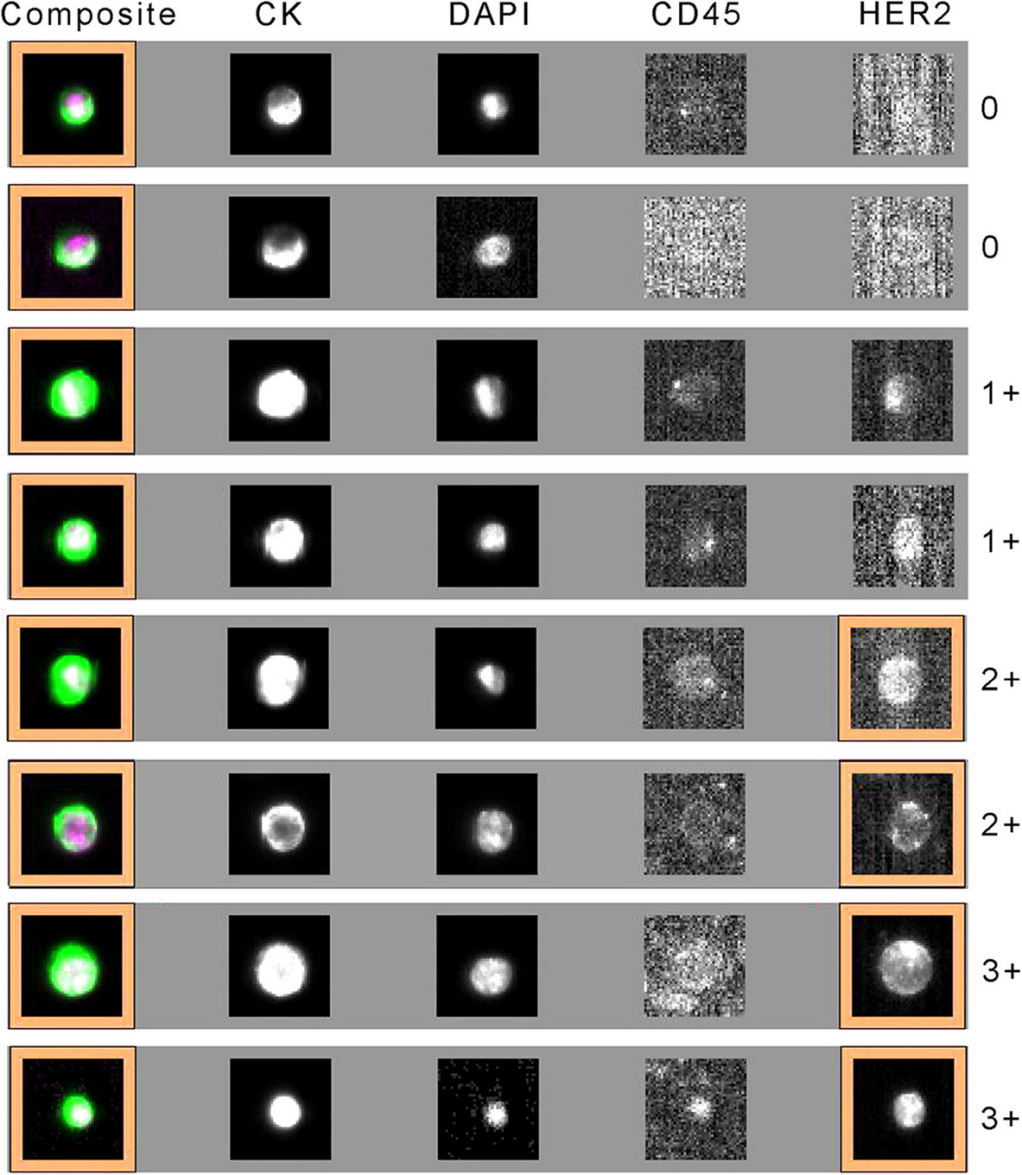
Representative images for the 0, 1+, 2+, and 3+ intensities of HER2 expression on CTCs.

HER2 expression intensity in CTCs was given a score of 0, 1+, 2+, or 3+, according to the criteria described previously [28,29], and representative images are shown in Figure 2. Additional file 2: Table S2 presents the percentages of CTCs at given HER2 intensity scores in both the HER2-positive and HER2–negative groups. With the positive criterion defined as >30% of CTCs over-expressing HER2 (3+), the positive and negative coincidence rates of CTC HER2 were 48% (13/27) and 100% (9/9), respectively, compared with tumor tissue. McNemar’s test demonstrated that the HER2 status of CTCs was significantly different from that of tumor tissues (Table 2, χ^2^ = 12.07, *P* = 0.0005).

**Table 2:** Comparison of HER2 status between tumor tissue and CTCs

**CTC HER2**	**Tumor tissue HER2**		**Total**
	**+**	**-**	
3+ >30%	13	0	13
3+ ≤30%	14	9	23
Total	27	9	36

χ^2^ = 12.07, *P* = 0.0005.

### HER2 Expression in CTCs as a tool for predicting anti-HER2 therapy efficacy

**Figure 3:**
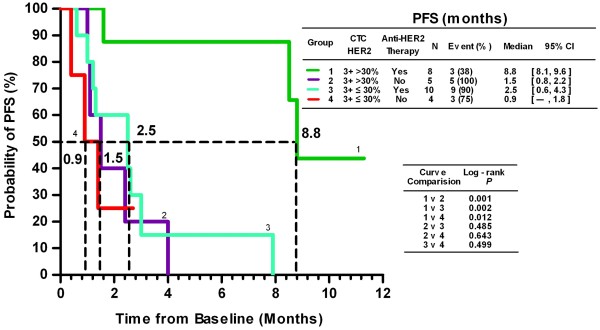
**Kaplan-Meier PFS plots of patients who have >30% or ≤30% of their CTCs with an HER2 intensity score of 3+, with or without anti-HER2 therapy.** PFS was calculated from the time of the baseline blood draw. Coordinates of the dashed lines indicate median survival time.

Twenty-seven patients with a CTC count ≥1 were divided into 4 groups based on their CTC HER2 status and whether they were receiving anti-HER2 therapy. Groups 1 and 2 consisted of patients with HER2 3+ CTC >30%, and groups 3 and 4 consisted of patients with HER2 3+ CTC ≤30%. Although all patients were histologically positive for HER2 and therefore should have received anti-HER2 therapy, patients in groups 2 and 4 did not receive the treatment for economic reasons. Kaplan-Meier plots of the PFS values for all of the groups are shown in Figure 3. Statistical analysis demonstrated that among the patients who received anti-HER2 therapy (N = 18, groups 1 and 3), only those with HER2-positive CTCs have benefited (8.8 vs. 2.5 months, *P* = 0.002). Among the patients with HER2-positive CTCs (N = 13, groups 1 and 2), the median PFS for those receiving anti-HER2 therapy was significantly longer than that for those without anti-HER2 therapy (8.8 vs. 1.5 months, *P* = 0.001). Notably, up to 52% (14/27) of the patients who were histologically assessed as HER2-positive had HER2-negative CTCs (N = 14, groups 3 and 4), and anti-HER2 therapy did not significantly improve the median PFS for these patients (2.5 vs. 0.9 months, *P* = 0.499). In addition, we also compared the PFS of HER2 3+ >10% vs. < 10%, HER2 3+ vs. HER2 (2+ and 1+) as well as HER2 (3+ and 2+) vs. HER2 (1+ and 0), but found no significant difference (data not shown).

## Discussion

In this study, we found that CTC enumeration with a cut-off of ≥1 but not ≥5 CTCs could serve as a useful prognostic factor for HER2-positive MBC patients. CTC enumeration using CellSearch (with a cut-off of ≥5 CTCs) is widely accepted as a prognostic factor for MBC patients [14-17]; however, its prognostic power for the HER2-positive subgroup seems to be inadequate [20]. More studies are needed to determine an appropriate cut-off for all subgroups of MBC patients. We found that HER2-positive patients had relatively lower CTC counts than ER-positive/HER2-negative patients. Our results were consistent with the report of Giordano et al., in which a larger proportion of HR-positive/HER2-negative patients had ≥5 CTCs than those with other tumor subtypes (*P* = 0.024) [20]. In line with these findings, Punnoose et al. found that the CTCs in a population of HR-positive/HER2-negative patients displayed higher levels of EpCAM, a CTC enrichment marker used in the CellSearch system [19]. It is possible that the ≥5-CTCs cut-off is an unsuitable prognostic indicator for a subgroup of HER2-positive patients. We tried other possible cut-offs and eventually found that the cut-off of ≥1 CTC yielded significant differences in PFS. Our results indicated that the underlying molecular subtype and gene expression patterns might not be same for different subtype of patients and that an adapted cut-off should be considered to make prognosis judgments for various subgroups of patients.

Based on clinical outcomes, our results indicated that a CTC HER2-positive criterion defined as >30% of CTCs over-expressing HER2 could improve the response prediction in anti-HER2 therapy. In recent years, great efforts have been undertaken to compare the HER2 status of tumor tissue and CTCs and determine whether anti-HER2 therapy would be beneficial. The studies summarized in Additional file 4: Table S3 indicated that the HER2 status of the CTCs was totally different from that of the tumor tissue. The overall discrepancy rate between the two sample sources ranged from 15% to 61%. More importantly, the HER2 detection methods used in these studies varied, and there is no current consensus on how HER2 positivity should be determined in CTCs.

For the IF-based HER2 staining method used in the CellSearch system, two HER2 positivity criteria were proposed. According to Pestrin et al., CTCs can be defined as HER2-positive if at least 50% of them were HER2-positive by IF [27]. Riethdorf et al. [28] and Ignatiadis et al. [29] noted that the intensity of HER2 staining using the CellSearch system was variable, ranging from absent or weak to intermediate and sometimes bright. They proposed a model in which HER2 expression in CTCs was scored as 0, 1+, 2+, or 3+ according to the staining intensity of HER2 in 6 types of breast cancer cell lines with known HER2 statuses [28,29]. CTCs were categorized as HER2-positive if at least one CTC showed strong HER2 staining intensity; however, due to CTC heterogeneity, the intensity of an individual CTC might not represent the actual HER2 status of the patient.

We postulated that a reasonable CTC HER2-positive criterion should seek experience from IHC, which detect HER2 protein expression with a similar technological principle as IF. Most importantly, the criterion should be validated by clinical evidence. The HER2 positive criterion using IHC was defined as uniform and intense membrane staining of >30% of invasive tumor cells membrane staining (the original threshold was >10%) [30]. Accordingly, we proposed and tested two criteria for HER2 positivity: >30% or >10% of CTCs over-expressing HER2. Conceivably, such criteria that combine qualitative and quantitative aspects encompassed a comprehensive evaluation of the entire pool of the isolated CTCs. Based on the patients’ clinical outcomes, we found that only the 30% threshold could give more precise instruction for anti-HER2 therapy.

Using this threshold, we found that, surprisingly, only patients who have both HER2-positive tumor tissue and CTCs could substantially benefit from anti-HER2 therapy. Conversely, up to 52% (14/27) of the histologically HER2-positive patients had actually HER2-negative CTCs, and these patients may not benefit from anti-HER2 therapy. Our results are consistent with the recent work of Niikura et al., who reported that patients with HER2-positive primary breast tumors could not benefit from trastuzumab therapy due to loss of HER2 in the metastases [11].

Our data underscore the importance and urgency of HER2 testing in CTCs, which is a real-time and dynamic procedure compared with HER2 testing on metastatic tumors. Through CTC characterization, patients with HER2-positive tumors and CTCs are strongly recommended to undergo anti-HER2 therapy. Furthermore, patients who have HER2-positive tumors but HER2-negative CTCs could avoid overtreatment with anti-HER2 agents.

Even though our study may help select patients for anti-HER2 therapy, it was an exploratory single-center study, and the number of the enrolled patients was not adequate for powerful statistical analysis. To obtain more robust evidence, large cohort, multi-center and prospective clinical trials should be designed in the near future, in which therapeutic decisions are based on HER2 analyses of both tumor tissue and CTCs.

## Conclusions

Our data demonstrate that CTC enumeration with a modified cut-off is a valuable prognostic tool for HER2-positive MBC patients. The HER2 status of CTCs may be different from that of tumor tissues and can predict responses to anti-HER2 therapy. Our findings underscore the necessity of a comprehensive CTC analysis (regarding both number and HER2 status), which may be a valuable prognostic and predictive tool for optimizing individually tailored therapies for HER2-positive MBC patients.

## Competing interests

The authors declare that they have no competing interests.

## Authors’ contributions

YL carried out the CTC analysis and wrote the manuscript. QL collected the clinical data and carried out the statistical analysis. TW, LB, SHZ and SKW collected blood and clinical data from the patients. HXH, SL and ZYH carried out the CTC analysis. YL, BL and ZFJ participated in the design and coordination of the study. All authors read and approved the final manuscript.

## Pre-publication history

The pre-publication history for this paper can be accessed here:

http://www.biomedcentral.com/1471-2407/13/202/prepub

## Supplementary Material

Additional file 1: Table S1 Pathological and Clinical Characteristics of HER2-Negative Patients at Baseline. Click here for file

Additional file 2: Table S2 The clinical data of patients who detected CTC and the intensity and percentage of HER2 expression on CTCs. Click here for file

Additional file 3: Figure S1 Kaplan-Meier PFS plots of HER2-positive MBC patients with a cut-off of ≥ 2 (A) and ≥3 or 4 (B) CTCs. PFS was calculated from the time of the baseline blood draw. Coordinates of dashed lines indicate median survival time. Click here for file

Additional file 4: Table S3 Previous literatures about HER2 status comparison between tumor tissue and CTCs. Click here for file
