# Supplementary material for: Circulating tumor cells in HER2-positive metastatic breast cancer patients: a valuable prognostic and predictive biomarker
Source: BMC Cancer. 2013 Apr 23;13:202. doi: 10.1186/1471-2407-13-202 (PMC3643882; doi:10.1186/1471-2407-13-202)
Supplement: Additional file 1: Table S1 — Pathological and Clinical Characteristics of HER2-Negative Patients at Baseline. [file 1471-2407-13-202-S1.doc]

| Characteristics | Total | No. patients (%) | | *P* | No. patients (%) | | *P* |
| --- | --- | --- | --- | --- | --- | --- | --- |
|  |  | CTC Count at baseline | |  | CTC Count at baseline | |  |
|  |  | ≥1 | ＜1 |  | ≥5 | ＜5 |  |
| **Overall** | 11 | 9 (81.8) | 2 (18.2) |  | 7 (63.6) | 4 (36.4) |  |
| **Age (years)** |  |  |  |  |  |  |  |
| Mean | 50.6 | 48.9 | 55.0 | 0.363 | 49.1 | 51.5 | 0.630 |
| Range | 36-63 | 35-58 | 48-63 |  | 35-58 | 43-63 |  |
| **Histology** |  |  |  |  |  |  |  |
| Ductal | 9 | 8 (88.9) | 1 (11.1) | 0.240 | 6 (66.7) | 3 (33.3) | 0.662 |
| Lobular | 0 | 0 (0.0) | 0 (0.0) |  | 0 (0.0) | 0 (0.0) |  |
| Others | 2 | 1 (50.0) | 1 (50.0) |  | 1 (50.0) | 1 (50.0) |  |
| **ER** |  |  |  |  |  |  |  |
| Positive | 10 | 8 (80.0) | 2 (20.0) | 0.515 | 6 (60.0) | 4 (40.0) | 0.327 |
| Negative | 1 | 1 (100.0) | 0 (0.0) |  | 1 (100.0) | 0 (0.0) |  |
| **PR** |  |  |  |  |  |  |  |
| Positive | 8 | 6 (75.0) | 2 (25.0) | 0.231 | 4 (50.0) | 4 (50.0) | 0.068 |
| Negative | 3 | 3 (100.0) | 0 (0.0) |  | 3 (100.0) | 0 (0.0) |  |
| **No. of Metastasis** |  |  |  |  |  |  |  |
| 1 | 1 | 1 (100.0) | 0 (0.0) | 0.515 | 0 (0.0) | 1 (100.0) | 0.138 |
| ≥2 | 10 | 8 (80.0) | 2 (20.0) |  | 7 (70.0) | 3 (30.0) |  |
| **Metastatic sites** |  |  |  |  |  |  |  |
| Bone only | 1 | 1 (100.0) | 0 (0.0) | 0.491 | 0 (0.0) | 1 (100.0) | 0.157 |
| Visceral only | 0 | 0 (0.0) | 0 (0.0) |  | 0 (0.0) | 0 (0.0) |  |
| Bone and visceral | 9 | 7 (77.8) | 2 (22.2) |  | 6 (66.7) | 3 (33.3) |  |
| **DFS** |  |  |  |  |  |  |  |
| ≤12 months | 1 | 1 (100.0) | 0 (0.0) | 0.491 | 1 (100.0) | 0 (0.0) | 0.383 |
| >12 months | 9 | 7 (77.8) | 2 (22.2) |  | 6 (66.7) | 3 (33.3) |  |

**Supplemental Table 1**. Pathological and Clinical Characteristics of HER2-Negative Patients at Baseline
