# Supplementary material for: Circulating tumor cells in HER2-positive metastatic breast cancer patients: a valuable prognostic and predictive biomarker
Source: BMC Cancer. 2013 Apr 23;13:202. doi: 10.1186/1471-2407-13-202 (PMC3643882; doi:10.1186/1471-2407-13-202)
Supplement: Additional file 2: Table S2 — The clinical data of patients who detected CTC and the intensity and percentage of HER2 expression on CTCs. [file 1471-2407-13-202-S2.doc]

|  | Patient No. | CTC count | 3+ (%) | 2+ (%) | 1+ (%) | 0 (%) | PFS (Month) | Treatment line | Anti-HER2 therapy  after blood draw |
| --- | --- | --- | --- | --- | --- | --- | --- | --- | --- |
| HER2 positive patients | 2 | 13 | 2 (15.4) | 3 (23.1) | 3 (23.1) | 5 (38.4) | 0.6 | ≥4 | YES |
|  | 4 | 1 | 0 | 1 (100) | 0 | 0 | 2.6 | 1 | YES |
|  | 5 | 1 | 0 | 0 | 0 | 1 (100) | 3.0 | 3 | YES |
|  | 6 | 1 | 0 | 0 | 0 | 1 (100) | 2.5 | 3 | YES |
|  | 7 | 2 | 1 (50) | 1 (50) | 0 | 0 | 8.8 | 3 | YES |
|  | 13 | 2 | 0 | 1 (50) | 1 (50) | 0 | 1.2 | 1 | YES |
|  | 14 | 1140 | 437 (38.3) | 281 (24.7) | 246 (21.6) | 176 (15.4) | 1.5 | ≥4 | NO |
|  | 15 | 159 | 62 (39.0) | 50 (31.5) | 42 (26.4) | 5 (3.1) | 11.3 | 1 | YES |
|  | 17 | 2 | 1 (50) | 0 | 0 | 1 (50) | 1.6 | 2 | YES |
|  | 18 | 28 | 12 (42.9) | 4 (14.3) | 8 (28.6) | 4 (14.3) | 8.5 | 1 | YES |
|  | 19 | 1 | 0 | 0 | 1 (100) | 0 | 0.9 | ≥4 | NO |
|  | 21 | 1 | 0 | 0 | 0 | 1 (100) | 0.4 | ≥4 | NO |
|  | 22 | 40 | 19 (47.5) | 13 (32.5) | 5 (12.5) | 3 (7.5) | 2.4 | ≥4 | NO |
|  | 24 | 1 | 0 | 1 (100) | 0 | 0 | 1.4 | 3 | NO |
|  | 26 | 9 | 5 (55.6) | 2 (22.2) | 1 (11.1) | 1 (11.1) | 1.1 | ≥4 | NO |
|  | 29 | 4 | 2 (50) | 1 (25) | 0 | 1 (25) | 10.3 | 1 | YES |
|  | 31 | 2 | 0 | 1 (50) | 1 (50) | 0 | 7.9 | ≥4 | YES |
|  | 37 | 185 | 19 (10.3) | 50 (27.0) | 46 (24.9) | 70 (37.8) | 1.3 | ≥4 | YES |
|  | 38 | 2 | 2 (100) | 0 | 0 | 0 | 1.0 | 3 | NO |
|  | 43 | 10 | 7 (70) | 3 (30) | 0 | 0 | 4.0 | 1 | NO |
|  | 46 | 5 | 0 | 0 | 1 (20) | 4 (80) | 2.5 | ≥4 | YES |
|  | 48 | 2 | 0 | 0 | 1 (50) | 1 (50) | 1.0 | 3 | YES |
|  | 49 | 32 | 15 (46.9) | 9 (28.1) | 3 (9.4) | 5 (15.6) | 6.3 | 3 | YES |
|  | 50 | 1 | 0 | 0 | 0 | 1 (100) | 2.9 | ≥4 | YES |
|  | 52 | 177 | 103 (58.2) | 39 (22.0) | 14 (7.9) | 21 (11.9) | 2.9 | 2 | YES |
|  | 53 | 9 | 0 | 1 (11.1) | 3 (33.3) | 5 (55.6) | 2.7 | ≥4 | NO |
|  | 54 | 2 | 1 (50) | 0 | 0 | 1 (50) | 2.7 | 1 | YES |
| HER2 negative patients  (No.1-9 were ER+) | 1 | 3 | 0 | 0 | 0 | 3 (100) | 1.7 | 2 | NO |
|  | 2 | 4 | 0 | 0 | 0 | 4 (100) | 1.3 | 3 | NO |
|  | 3 | 6099 | 0 | 21 (0.3) | 102 (1.7) | 5976 (98.0) | 1.3 | 3 | NO |
|  | 5 | 6 | 0 | 0 | 0 | 6 (100) | 0.9 | ≥4 | NO |
|  | 6 | 831 | 0 | 85 (10.2) | 185 (22.3) | 561 (67.5) | 2.6 | ≥4 | YES |
|  | 7 | 509 | 0 | 21 (4.1) | 96 (18.9) | 392 (77.0) | 1.2 | 3 | NO |
|  | 8 | 16 | 0 | 0 | 4 (25) | 12 (75) | 3.4 | ≥4 | NO |
|  | 9 | 212 | 0 | 13 (6.1) | 21 (9.9) | 178 (84.0) | 2.5 | 3 | NO |
|  | 11 | 36 | 0 | 0 | 2 (5.6) | 34 (94.4) | 1.0 | ≥4 | NO |

**Supplemental Table 2.** The clinical data of patients who detected CTC and the intensity and percentage of HER2 expression on CTCs
