# Supplementary figures and images for: Circulating tumor cells in HER2-positive metastatic breast cancer patients: a valuable prognostic and predictive biomarker
Source: BMC Cancer. 2013 Apr 23;13:202. doi: 10.1186/1471-2407-13-202 (PMC3643882; doi:10.1186/1471-2407-13-202)

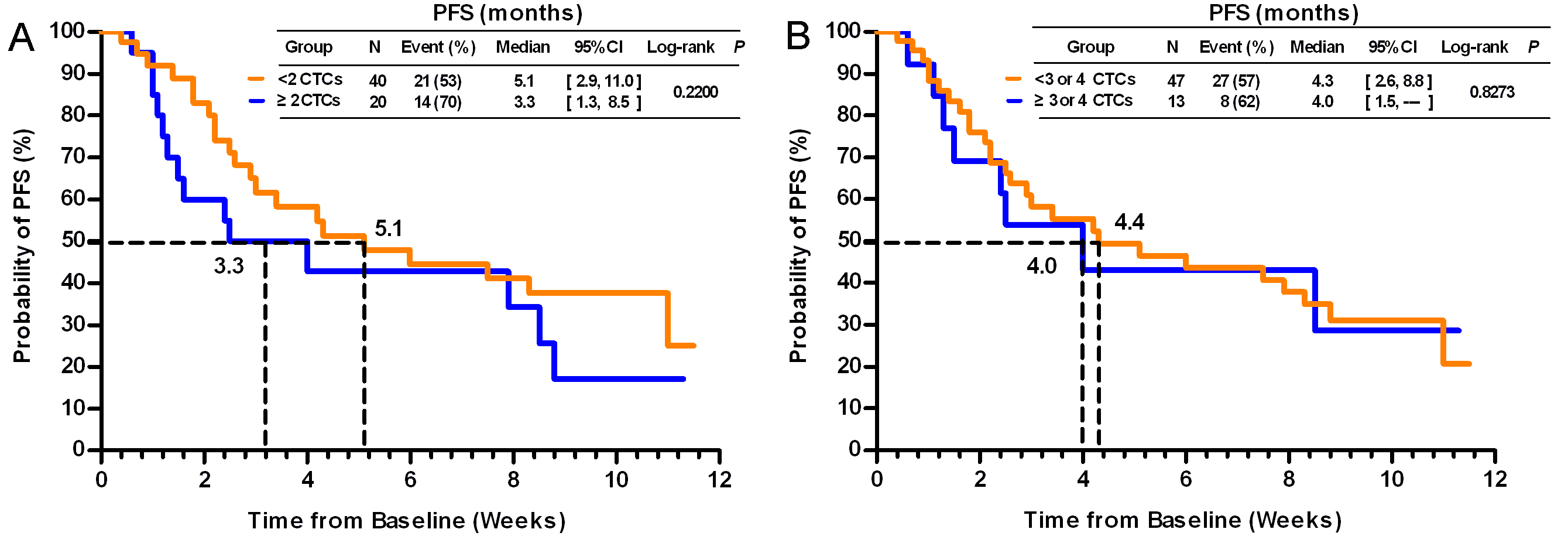

Supplement: Additional file 3: Figure S1 — Kaplan-Meier PFS plots of HER2-positive MBC patients with a cut-off of ≥ 2 (A) and ≥3 or 4 (B) CTCs. PFS was calculated from the time of the baseline blood draw. Coordinates of dashed lines indicate median survival time. [file 1471-2407-13-202-S3.jpeg]
