# Supplementary material for: Circulating tumor cells in HER2-positive metastatic breast cancer patients: a valuable prognostic and predictive biomarker
Source: BMC Cancer. 2013 Apr 23;13:202. doi: 10.1186/1471-2407-13-202 (PMC3643882; doi:10.1186/1471-2407-13-202)
Supplement: Additional file 4: Table S3 — Previous literatures about HER2 status comparison between tumor tissue and CTCs. [file 1471-2407-13-202-S4.doc]

| Ref | Disease setting | Method of CTC isolation  and HER2 evaluation | HER2-positive criterion for CTC | HER2 discordant rate | | |
| --- | --- | --- | --- | --- | --- | --- |
|  |  |  |  | Overall | Tumor+ / CTC- | Tumor- / CTC+ |
| 21 | MBC | CELLection Dynabeads, RT-PCR | HER2 expression was detected | 39% (10/26) | 31% (4/13) | 46% (6/13) |
| 22 | MBC | *AdnaTest BreastCancer.* | HER2 expression was detected | 36% (8/22) | 60% (3/5) | 29% (5/17) |
| 23 | MBC | A: CellSearch, IF  B: AdnaTest Breast Cancer | A: at least one CTC was 3+  B: HER2 expression was detected | A: 36% (38/107)  B: 52% (41/79) | A: 42% (13/31)  B: 59% (13/22) | A: 33% (25/76)  B: 49% (28/57) |
| 24 | MBC | A: Slide based assay and FISH  B: AdnaTest Breast Cancer test | A: HER-2/CEP 17≥2  B: HER2 expression was detected | 40% (6/15) | 67% (2/3) | 33% (4/12) |
| 25 | Stage I to III | Immunomagnetic separation,  immunocytochemistry | Strong complete membrane staining in >10% of cells. | NA | NA | 12 patients |
| 18 | MBC | CPK of CellSearch, FISH | HER-2/CEP 17≥2 | 15% (11/75) | 2% (1/45) | 33% (10/30) |
| 26 | Stage Ⅰ-Ⅳ | Immunomagnetic enrichment, FISH | HER-2/CEP 17≥2 | 31% (13/42) | 27% (4/15) | 33% (9/27) |
| 27 | ABC | CellSearch, IF | At least 50% of CTCs were HER2 positive by IF | 33% (13/40) | 42% (5/12) | 29% (8/28) |
| 28 | Neoadjuvant | CellSearch, IF | At least one CTC showed moderate or strong HER2 IF | 32% (12/37) | 46% (5/11) | 27% (7/26) |
| 29 | DCIS/LCIS, M0 (A) and M1 BC | CellSearch, IF  (A: 22.5ml M1: 7.5ml) | HER2 Intensity that is at least 2.5 higher than the background. | A: 29% (5/17)  M1: 61% (14/23) | A: 0% (0/5)  M1: 50% (1/2) | A: 42% (5/12)  M1: 62% (13/21) |
| 19 | MBC | CellSearch, IF | A weighted score based on the number of CTCs with a given level of expression. | 17% (5/29) | 18% (2/11) | 17% (3/18) |

**Supplemental Table 3**. Previous literatures about HER2 status comparison between tumor tissue and CTCs
